# Addressing the obstacles in cultivating global advocates for traditional Chinese medicine: qualitative perspectives from stakeholder interview analysis

**DOI:** 10.3389/fpubh.2025.1703208

**Published:** 2025-11-18

**Authors:** Xi Li, Ji Chen, Qin Li, Fuyuan Lei, Ping Yi

**Affiliations:** School of Foreign Languages, Chengdu University of Traditional Chinese Medicine, Chengdu, China

**Keywords:** international dissemination of TCM, dilemmas, stakeholders, dual-track knowledge translation, collaborative governance

## Abstract

**Background:**

In the context of accelerated globalization of traditional Chinese medicine (TCM), cultural disparities and heterogeneity in cognitive frameworks have intensified dissemination resistance, resulting in multidimensional challenges in talent cultivation. Delays in terminological translation, cross-cultural communication barriers, ambiguous career pathways, coupled with uneven resource allocation and policy misalignment, significantly hinder the effectiveness of TCM’s international promotion.

**Methods:**

Between May and June 2025, semi-structured in-depth interviews were conducted with four stakeholder groups—students, educators, scholars, and experts (*N* = 31)—focusing on core challenges in international talent development, role perception, resource coordination bottlenecks, and strategic intervention measures. The analysis employed a “Micro-Meso-Macro” framework to systematically examine interactions among individual practices, pedagogical system restructuring, and institutional tensions.

**Results:**

The findings reveal that students and educators primarily emphasize linguistic and pedagogical barriers, whereas scholars and experts underscore deficiencies in interdisciplinary paradigms and institutionalized training mechanisms. The central contradiction involves the disconnect between international talent demands and localized educational systems, characterized by homogenized talent cultivation and inadequate adaptation to cross-cultural competencies and international standards. Institutional bottlenecks include short-term resource investments and the lack of effective coordination mechanisms, leading to neglect of foundational infrastructure over the long term.

**Conclusion:**

An innovative “dual-track knowledge translation and collaborative governance” framework is proposed. Top-level design should involve government-led establishment of an international TCM dissemination alliance and dedicated funding to enhance infrastructure; universities should develop cross-cultural curricula and a national terminology standard platform, integrated into professional certification systems; mechanism innovation should focus on establishing core competency standards for TCM internationalization, aligning stakeholder interests, and fostering sustainable, ecosystem-based development.

## Highlights

1. Presented the inaugural theoretical model of the “Dual Track System of Knowledge Translation and Collaborative Governance.”
2. Executed comprehensive, multi-perspective qualitative interviews and discourse analyses to identify obstacles to the global dissemination of Traditional Chinese Medicine.
3. Findings indicate that both students and educators exhibit significant concern regarding linguistic and conceptual barriers within pedagogical frameworks.
4. Academic scholars and domain experts predominantly emphasize deficiencies in interdisciplinary paradigm integration and the formalization of training protocols.

## 1 Introduction

Despite the predominance of Western biomedicine within the global health paradigm, its rapid advancement during the early 20th century has achieved substantial international recognition (1). Traditional Chinese Medicine (TCM), as an integrative medical system with a history exceeding a millennium, is progressively attaining unprecedented global acknowledgment (2). According to data from the World Health Organization (WHO), 170 member states incorporate traditional and complementary medicine into their healthcare frameworks (3), with Chinese herbal medicine disseminated across 196 countries and regions. Acupuncture is endorsed by 113 WHO member states (4), and Chinese medicine is increasingly regarded as a vital healthcare resource and cultural symbol (5). Nonetheless, despite the accelerating globalization of TCM, its cross-cultural dissemination exemplifies a complex interplay between traditional paradigms and global integration (6), confronting numerous challenges such as conceptual misinterpretations, fragmented educational infrastructures, and inconsistent international credentialing standards (7). These issues are rooted not only in linguistic barriers but also deeply embedded within philosophical paradigms, cognitive schemas, and institutional frameworks.

A review of extant scholarly literature indicates that research on the internationalization of TCM predominantly emphasizes translation fidelity (8) and clinical efficacy (9, 10), often neglecting the multi-stakeholder dynamics that influence the development of competent TCM practitioners. Most investigations adopt linguistic or biomedicine-centric perspectives, insufficiently addressing how cultural adaptation, pedagogical strategies, and policy environments interact to impact educational outcomes. The knowledge translation framework (11), extensively utilized in global health to bridge research and practice, remains underexplored within the context of TCM. Similarly, the application of collaborative governance theory, which has demonstrated efficacy in managing complex public health initiatives (12), has yet to be systematically examined regarding its capacity to coordinate diverse stakeholders in the international education and credentialing of TCM practitioners.

This research gap holds considerable significance within the domain of traditional medicine globalization. On one hand, the deficiency in effective intercultural competency training leads to the reduction of core Traditional Chinese Medicine (TCM) concepts—such as “Qi”, "Yin-Yang” and the “Five Elements”-to metaphysical symbols or overly simplified biomedical analogies (13), thereby compromising their epistemological validity and clinical efficacy. On the other hand, discrepancies between domestic curricula and international accreditation standards often necessitate redundant overseas training for graduates; additionally, inadequate intercultural competence may result in patient mistrust and resistance to policy implementation (14). If these issues remain unaddressed, the global legitimacy of TCM could be undermined, and its capacity for intercultural dialogue and knowledge exchange may be impeded. Consequently, this study primarily investigates the following research questions (RQ) (Table 1).

**Table 1:** Research questions.

NO.	Type 1	Type 2	Research questions
1	RQ1	Micro	How can terminological translation facilitate semantic negotiation and cultural adaptation to resolve cognitive conflicts in the international dissemination of TCM, thereby enhancing learner identification and intercultural communication confidence?
2	RQ2	Meso	In the development of TCM international educators, how can knowledge translation through pedagogical strategy and assessment reform better accommodate diverse cultural audiences?
3	RQ3	Macro	Under multi-stakeholder governance, how can regulatory frameworks, incentive structures, and accountability mechanisms be optimized to reshape the talent cultivation system for the global promotion of TCM?

**Figure 1:**
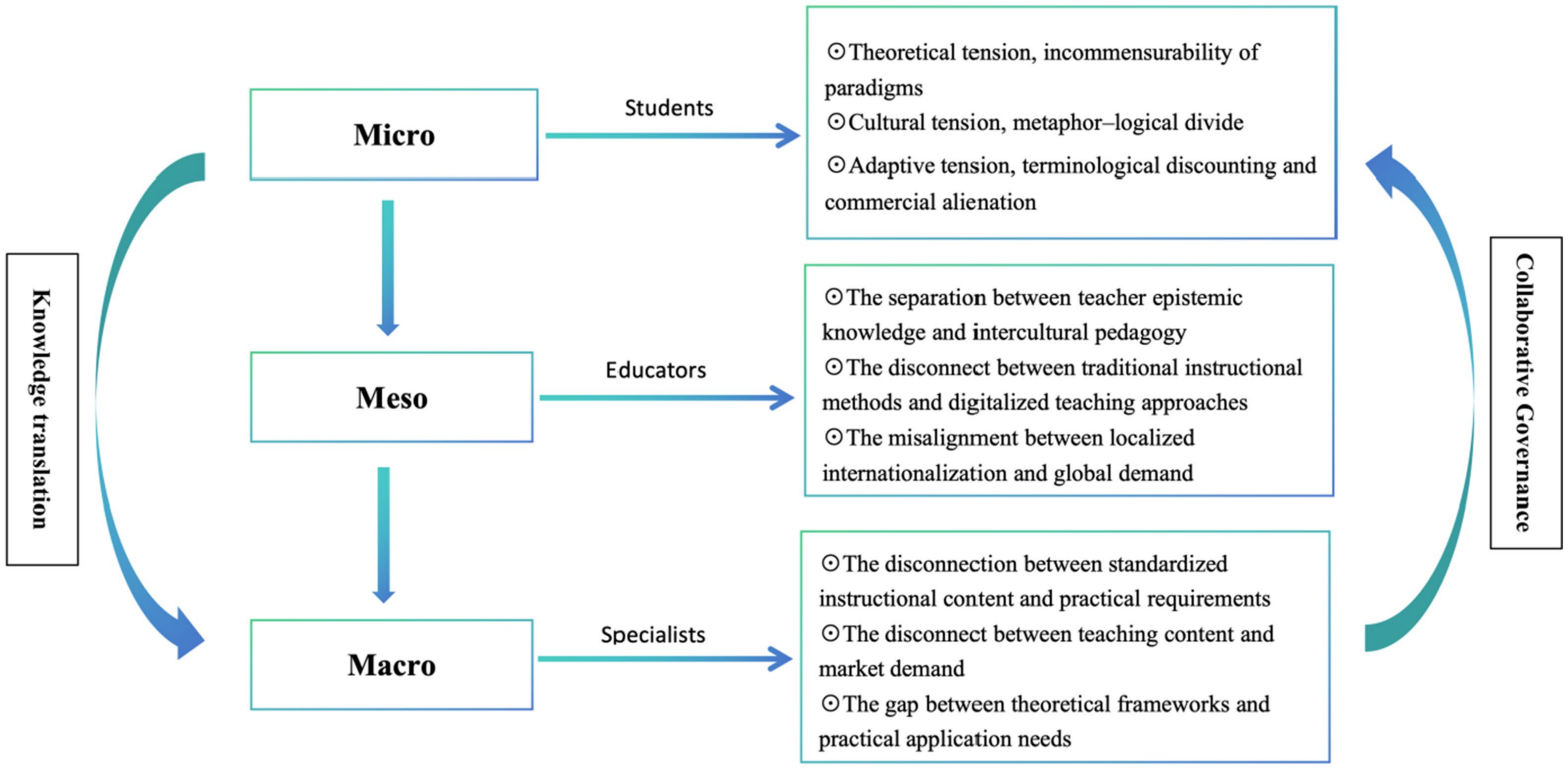
Micro-Meso-Macro multi-level analytical framework diagram.

This study systematically examines challenges across micro, meso, and macro levels, aiming to cultivate TCM ambassadors with a comprehensive global perspective. Drawing on theories of knowledge translation and collaborative governance (11, 12), and integrating insights from key stakeholders—including students, educators, practitioners, and scholars—a multi-layered analytical framework is developed (Figure 1). Through 31 semi-structured interviews, specific barriers encountered by each stakeholder group are identified, revealing cross-level contradictions that hinder cultural authenticity, pedagogical effectiveness, and alignment with international qualification standards. This study aims to promote a deeper academic understanding of the globalization of TCM, aligning with the policy context of the World Health Organization’s “Global Strategy for Traditional Medicine 2025–2034” and conducting in-depth research on the key issue of “mismatch between talent supply and international demand” in the globalization process of TCM. Simultaneously, it provides theoretical and practical references for how traditional medical systems can achieve genuine and effective integration within a diversified healthcare landscape.

## 2 Literature review and theoretical framework

### 2.1 Cultural translation and global health communication

Intercultural medical communication is fundamental for practitioners involved in global health initiatives (15), encompassing not only linguistic equivalence but also the negotiation of epistemological frameworks and symbolic systems (16). Cultural translation pertains to the process of conveying complex, multilingual, and cross-cultural contexts while preserving conceptual fidelity and adapting to the cognitive schemas of the target audience (17). Cultural mediators with specialized insights are particularly vital. Within the scope of global health, cultural translation encounters substantial challenges, especially when divergent medical paradigms are rooted in fundamentally different ontologies—such as the holistic and phenomenological principles of TCM contrasted with the reductionist, evidence-based approach of Western biomedicine—necessitating ongoing refinement in translation accuracy, semantic fidelity, and cultural validation (18).

For instance, in TCM, concepts like Qi, Yin-Yang, and the Five Elements represent dynamic interactions between the human body, environmental factors, and the cosmos (19). Translating these specialized constructs demands effective cultural adaptation to prevent misinterpretation as mystical abstractions or overly simplified biomedical analogies (20), which could undermine their clinical significance and depth (21). Current research underscores that precision in cultural translation is critical for the global dissemination of TCM, as it directly impacts the accurate transmission of its theoretical foundations and influences its international acceptance and legitimacy (22). Consequently, translators engaged in international TCM education must possess robust linguistic competencies alongside a deep understanding of TCM’s philosophical and cultural underpinnings to ensure translation fidelity and conceptual depth. As global health communication functions as a pivotal conduit for TCM’s internationalization, the development and implementation of innovative dissemination strategies and methodologies are imperative (23).

### 2.2 Knowledge translation in cross-cultural medical education

Amidst globalization, the internationalization of higher education institutions is progressively advancing. Universities are expanding international exchange programs through global talent recruitment, joint degree initiatives, and collaborative research partnerships, thereby increasing opportunities for international academic mobility and faculty exchange. Emphasis is placed on understanding and enhancing the internationalization experience of students (24). Within the framework of global health governance, fostering cross-cultural exchanges in TCM and promoting the international development of TCM higher education are of paramount importance (20). Therefore, intercultural competence of teachers and students is necessary (25, 26). The concept of intercultural competence is a combination of cognition, emotion and behavior. When these three are integrated or applied collectively among professionals, they can work effectively in an intercultural environment. Intercultural competence is also one of the key competencies of international talents in the context of globalization (27, 28).

The knowledge translation (KT) framework, extensively utilized in evidence-based medicine, provides a systematic methodology to bridge the gap between research evidence and clinical implementation (29). The boundary between knowledge generation and application is dynamic; new scientific insights can inform clinical practice, and feedback from implementation processes can lead to the refinement of existing knowledge or the emergence of novel concepts. This bidirectional interaction exemplifies the reciprocal influence inherent in KT (30). Knowledge translation involves enhancing individual, organizational, and systemic capacities to convert high-quality clinical theories into practical applications (31). The framework emphasizes contextual adaptation, iterative feedback mechanisms, and stakeholder engagement, making it particularly pertinent to the internationalization of TCM, where scientific validation must be balanced with cultural preservation.

Current research indicates that, within a globalized and multicultural society, international collaboration continues to reshape medical education and clinical practice, fostering equitable and inclusive cross-cultural medical training (32). However, within the KT paradigm, the international dissemination of TCM necessitates a dual focus: first, content adaptation to ensure that core medical principles are both accurate and accessible to international learners; second, continuous refinement of pedagogical strategies, clinical protocols, and assessment standards to accommodate diverse learning styles and cultural contexts. This approach aims to better serve heterogeneous learner populations and to address the complexities of cross-cultural educational environments (33). Despite the critical role of KT in global health education, its application to the internationalization of TCM remains limited (34). Existing literature often perceives the international teaching of TCM as a unidirectional transfer of knowledge, neglecting the co-constructive process of meaning-making between educators and learners from diverse cultural backgrounds. Such oversight hampers translation efficiency, results in fragmented curriculum design, and creates misalignments with international accreditation standards.

### 2.3 Collaborative governance and stakeholder engagement

The development of TCM professionals with international communication competencies involves a complex, multi-stakeholder collaborative process among government agencies, academic institutions, industry associations, enterprises, healthcare providers, and international partners (35, 36). According to theories of collaborative governance, sustained and structured multi-stakeholder participation is critical for effective governance in intricate policy environments (37). In the international dissemination of TCM, different stakeholders have varying priorities. Governments emphasize cultural diplomacy and building national soft power; universities focus on academic standards, international accreditation, and research output; medical institutions prioritize clinical efficacy and patient safety; and international partners are concerned with local medical regulations and patient expectations. If these priorities are not effectively aligned at the institutional level, it often leads to a disconnect between top-level design and implementation, and insufficient policy coordination. Furthermore, unclear resource allocation and division of responsibilities at the policy implementation level can easily result in inconsistent training standards and low efficiency. Therefore, insufficient resource allocation manifests both as a lack of top-level coordination in institutional design and as a failure of collaboration at the policy implementation level. Collaborative governance, through cross-departmental and cross-institutional co-construction mechanisms and multi-dimensional collaboration from institutional to implementation perspectives, has become an effective path to ensure the international dissemination of TCM and guarantee the quality of the teaching process. Literature reviews reveal that current research predominantly concentrates on joint training initiatives within academic settings, emphasizing the integration of scientific research and education (38). the adoption of digital technologies and artificial intelligence (39), and the promotion of interdisciplinary collaboration (40). Through collaborative governance, stakeholders can collectively participate in cultivating internationally competent TCM practitioners, fostering synergistic efforts to improve training outcomes—an effective mechanism for stakeholder coordination. Nonetheless, empirical applications of such models in international TCM education are limited, with notable deficiencies in theoretical guidance and practical implementation strategies.

### 2.4 Cultural translation, knowledge transformation, and the theoretical framework of collaborative governance

**Figure 2:**
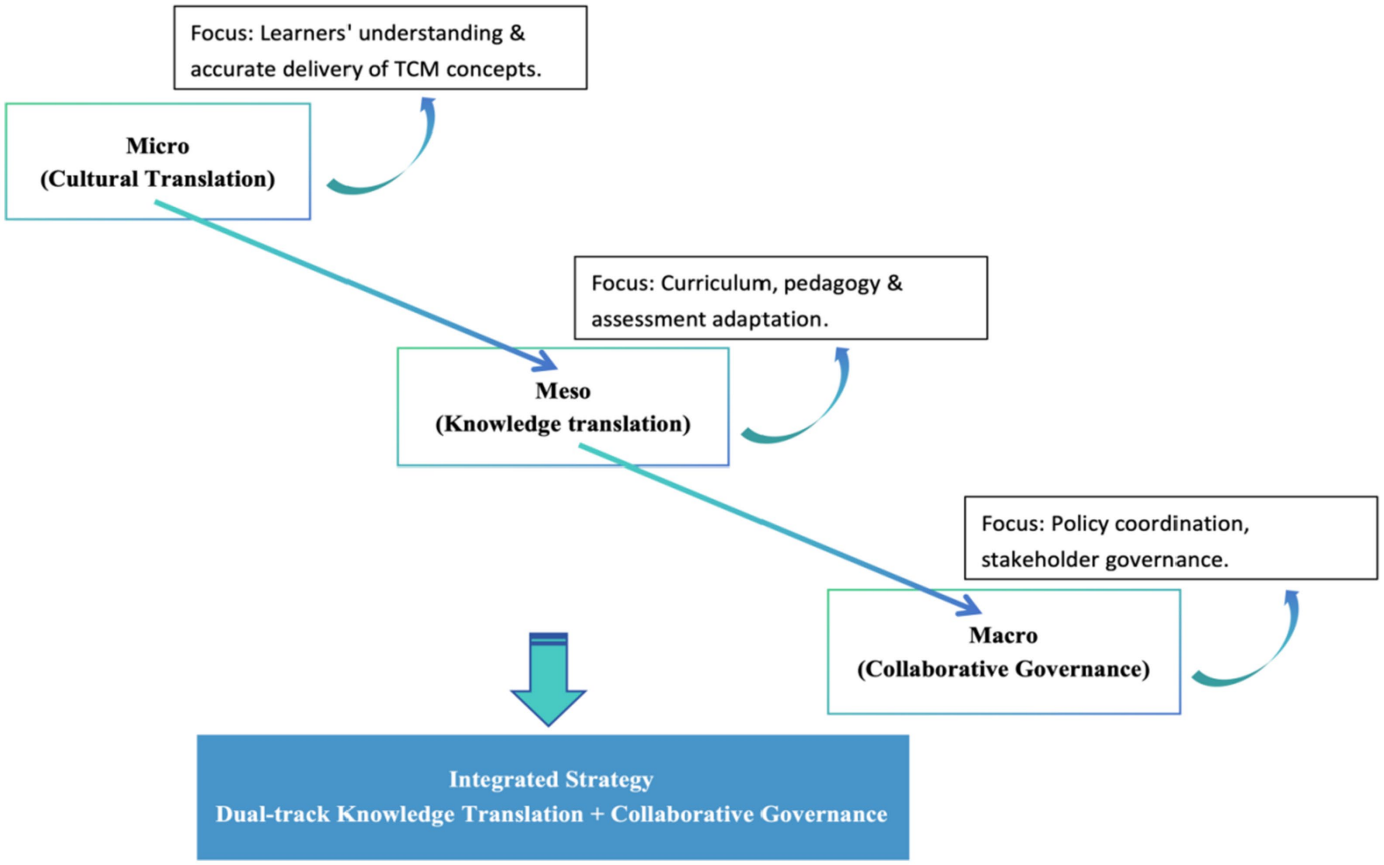
Schematic of dual-track knowledge transformation and collaborative governance.

Building upon existing literature, this study synthesizes cultural translation, knowledge transformation, and collaborative governance within a multi-level analytical framework (41), establishing a three-dimensional model—micro, meso, and macro—to examine the cultivation of talent for international TCM dissemination. Specifically, at the micro level, cultural translation pertains to individual learners’ capacity to comprehend and accurately communicate TCM concepts. At the meso level, knowledge transformation involves adapting curricula, pedagogical approaches, and assessment systems through institutional strategies to accommodate diverse cultural and educational contexts. At the macro level, collaborative governance emphasizes policy and structural mechanisms designed to coordinate stakeholder relationships and facilitate systemic reforms. This integrated framework enables comprehensive analysis of barriers and facilitators and provides a foundation for developing a “dual-track knowledge transformation coupled with collaborative governance” strategy (Figure 2). Such an approach aims to preserve the integrity of TCM knowledge and theoretical frameworks while enhancing pedagogical flexibility and ensuring regulatory compliance, thereby addressing the diverse needs of the global healthcare landscape.

## 3 Method

### 3.1 Research design

This investigation adopts a qualitative research paradigm, emphasizing social science inquiry aimed at identifying recurrent thematic patterns and their interrelations within individual perceptions articulated through language and communicative acts (42). Purposive maximum variation sampling was employed to select participants for semi-structured interviews (43), capturing diverse perspectives, experiential narratives, and strategic approaches from students, educators, scholars, and domain experts concerning the obstacles encountered in the international dissemination of TCM. Through comprehensive in-depth interviews, the study seeks to elucidate specific challenges in the global promotion of TCM, aiming to attain an in-depth understanding of participants’ subjective experiences and meaning-making processes, including those of students, instructors, researchers, and specialists.

### 3.2 Theoretical basis and question design of the interviews

The interview question design in this study is supported by the theories of “knowledge transformation” and “collaborative governance.” “Knowledge transformation” reveals the logic of knowledge dissemination at the content level, while “collaborative governance” analyzes the coordination mechanisms at the institutional level. It explores the flow of knowledge, role interaction, and institutional synergy in the international dissemination of TCM. Based on the theory of knowledge transformation, the interview questions first focus on the transformation path of TCM knowledge from academic production to cross-cultural application, involving how students and teachers understand and reconstruct TCM knowledge to adapt to international education and medical environments. For example, questions for students revolve around cultural understanding barriers, knowledge re-expression methods, and the translatability of local experiences encountered in learning and practice; teacher interviews focus on the strategies adopted in the selection of teaching content and knowledge localization. Simultaneously, the theory of collaborative governance emphasizes trust building, resource sharing, and joint decision-making mechanisms among stakeholders. Therefore, to delve deeper into the issues, our interview questions include: students’ learning motivation and expectations, learning difficulties and contradictions, future development and reality, scholars’ views on knowledge translation and discourse power, resources and support, and experts’ suggestions on the contradictions between standards and certification.

### 3.3 Ethical approval

To ensure the standardization and scientific rigor of the research process, this study strictly adhered to the principles outlined in the Declaration of Helsinki (44). Prior to commencement, a comprehensive ethical review application was submitted to the Ethics Review Committee of the XXXX University (Anonymous). The application detailed the study’s objectives, methodology, potential risks, and measures for safeguarding participants’ rights and interests. Following rigorous review and discussion, the committee approved the proposal, issuing an ethical approval document (Approval No.: 2005026).

### 3.4 Interviewers and reflexivity

Data collection was conducted by Xi Li and Ji Chen, both of whom possess academic, research, and practical expertise in higher education medical training and have received specialized training in qualitative interviewing techniques. Throughout the interview process, the interviewers maintained neutrality and objectivity, engaging in reflexive practices to evaluate their influence on data integrity (45). When addressing sensitive topics, interviewers carefully calibrated their questioning strategies to minimize leading responses and ensure the authenticity and objectivity of the data collected (46).

### 3.5 Participants

Participants were recruited via purposive maximum variation sampling (43), with data collection occurring between May 15 and June 10, 2025. A total of 31 individuals from diverse academic and professional backgrounds—including students, scholars, experts, and educators—were invited to participate. The students’ academic qualifications include undergraduate and graduate students; the scholars’ majors include linguistics, TCM, Chinese literature, etc.; and the experts include experts in the fields of TCM, traditional Chinese pharmacology, TCM culture, and international dissemination of TCM culture. The sample was intentionally heterogeneous to enhance the comprehensiveness and representativeness of the findings. Participant demographic details are summarized in Table 1. All participants provided informed consent, having been thoroughly briefed on the study’s aims and potential implications (47). Basic demographic data such as age, gender, academic credentials, and years of professional experience were recorded and anonymized during subsequent analysis.

### 3.6 Data collection

The semi-structured interview outlines for this study were reviewed by five experts from four research fields: TCM, Communication Studies, Foreign Languages and Literature, and Traditional Chinese Materia Medica. All five experts held the title of professor. The outlines were finalized after two pre-interviews prior to the formal interviews (Appendix). Each interview lasted approximately 30 to 45 min. The interview guide was systematically designed to encompass multiple dimensions, including motivational factors and expectations for learning, encountered difficulties and conflicts, curriculum design and pedagogical strategies, students’ competencies and aspirations, cross-cultural communication concepts, interdisciplinary training conflicts, market demand, and training objectives. All interviews were audio-recorded to ensure data fidelity, with verbatim transcription performed to capture the full scope of dialogue, facilitating detailed subsequent qualitative analysis.

### 3.7 Data saturation

During the qualitative data collection phase, we conducted iterative interviews until reaching data saturation, defined as the point at which subsequent interviews no longer generated novel themes or concepts, and any additional information pertaining to existing themes was minimal (20). In practice, real-time thematic analysis was performed during each interview, comparing emerging patterns with previously collected data. For instance, after 17 interviews with students, we observed that the following three interviews only reinforced existing themes, such as “Factors Influencing the International Dissemination of TCM” and “Challenges in the Global Spread of TCM,” without identifying new core themes. Following consensus and evaluation by the research team, we concluded that data saturation had been achieved and discontinued further data collection. This methodology ensured comprehensive and valid data acquisition while optimizing resource utilization.

### 3.8 Data analysis

In the data analysis process, this study adopted a three-level coding method to gradually extract the core themes related to the international dissemination of TCM (48). In the specific operation, Microsoft Excel was first used to preliminarily organize and classify the original interview data to ensure the systematization and traceability of the data; then, Nvivo 15 software was used to perform open, axial and selective coding on the text data, so as to realize the hierarchical abstraction and theoretical induction of the theme. Initially, open coding was applied to generate preliminary conceptual labels from interview transcripts. Subsequently, axial coding organized the data according to participant demographics and contextual factors. Finally, selective coding identified central themes and categories. To enhance analytical reliability and consistency, two independent researchers conducted the coding process. Throughout, particular emphasis was placed on contrasting coping strategies among diverse participant groups, integrating individual experiences and backgrounds to explore underlying determinants. Cross-group comparisons elucidated the complexity and heterogeneity of TCM’s international dissemination across varied social and cultural milieus.

## 4 Results

### 4.1 Participant demographic data

In terms of demographic variables, this study collected data on participants’ gender, age, educational qualification, and years of professional experience to thoroughly evaluate the potential confounding effects of these factors on the research findings during statistical analysis. The sample comprised five males and twenty-six females. The age distribution was as follows: 18 participants aged 19–29 years, three participants aged 30–39 years, eight participants aged 40–49 years, and two participants aged 50–59 years (Table 2). Regarding educational background, fifteen participants were undergraduate students (including six international students), two were master’s degree candidates, nine held master’s degrees, and five possessed doctoral degrees. In terms of professional roles, participants included seventeen students, fourteen educators, and practicing physicians. Concerning academic titles, there were three professors, six associate professors, and five lecturers (Table 3).

**Table 2:** Basic characteristics of research participants.

NO.	Category	Basic information	Frequency	Percentage
1	Sex	Male	5	16.13%
		Female	26	83.87%
2	Age	19–29	18	58.06%
		30–39	3	9.68%
		40–49	8	25.81%
		50–59	2	6.45%
3	Education background	Undergraduate	15	48.39%
		Master	11	35.48%
		Doctor	5	16.13%
4	Identity	Doctor/Teacher	14	45.16%
		Students	17	54.84%

**Table 3:** Basic information of the interviewee.

NO.	Identity	Sex	Education background	Title	Code
1	Student	Female	Master	/	S01
2	Student	Female	Undergraduate	/	S02
3	Student	Female	Undergraduate	/	S03
4	Student	Female	Undergraduate	/	S04
5	Student	Female	Undergraduate	/	S05
6	Student	Female	Master	/	S06
7	Student	Male	Undergraduate	/	S07
8	Student	Female	Undergraduate	/	S08
9	Student	Female	Undergraduate	/	S09
10	Student	Male	Undergraduate	/	S10
11	Student	Female	Undergraduate	/	S11
12	Student	Female	Undergraduate	/	S12
13	Student	Female	Undergraduate	/	S13
14	Student	Female	Undergraduate	/	S14
15	Student	Female	Undergraduate	/	S15
16	Student	Female	Undergraduate	/	S16
17	Student	Male	Undergraduate	/	S17
18	Teacher	Female	Doctor	Associate Professor	T01
19	Teacher	Female	Master	Associate Professor	T02
20	Teacher	Female	Master	Associate Professor	T03
21	Teacher	Female	Master	Lecturer	T04
22	Scholar	Female	Master	Lecturer	S01
23	Scholar	Female	Master	Associate Professor	S02
24	Scholar	Female	Doctor	Lecturer	S03
25	Scholar	Female	Doctor	Lecturer	S04
26	Scholar	Female	Master	Lecturer	S05
27	Specialist	Male	Master	Associate Professor	S01
28	Specialist	Female	Doctor	Professor	S02
29	Specialist	Male	Doctor	Professor	S03
30	Specialist	Female	Master	Associate Professor	S04
31	Specialist	Female	Master	Professor	S05

### 4.2 Coding results

Through a systematic and detailed qualitative analysis, we identified and synthesized five core thematic categories, each comprising several subthemes. These thematic constructs elucidate the key challenges and ethical considerations encountered by practitioners engaged in the international propagation of TCM. The results offer an in-depth perspective on the multifaceted obstacles faced in the global dissemination of TCM (Table 4).

**Table 4:** Thematic analysis of challenges in the international dissemination of traditional Chinese medicine talents.

NO.	Topic	Subtopic	Key points
1	Core contradictions	Differences in cultural understanding	The insufficient comprehension of the deeply embedded traditional cultural foundations of TCM impedes precise translation and interpretation. While literal translations of terminology are achievable, understanding of core philosophical constructs such as “Yin and Yang,” “Five Elements,” and “Qi” remains superficial, thereby obstructing the grasp of their underlying metaphysical significance. Variations in cultural connotations and inconsistent terminological standards further exacerbate this challenge.
		Conflicts in cognitive frameworks	The empiricist paradigm and linear diagnostic framework characteristic of Western medicine influence perceptions of TCM’s holistic diagnostic methodology. Students trained within Western biomedical paradigms tend to interpret TCM through this reductionist lens, which hampers a comprehensive understanding of its unique philosophical principles, including holistic observation and syndrome differentiation.
2	Cross-cultural communication	Cultural incommensurability	The intrinsic cultural essence of TCM, rooted in traditional Chinese philosophical thought, encounters fundamental cross-cultural transmission barriers. This is primarily due to the profound philosophical implications embedded within key terminologies. For learners lacking Confucian or Daoist backgrounds, these terms are often misconstrued as mystical symbols or material entities, thereby obscuring their dynamic relational and conceptual significance.
		Barriers to cultural comprehension	TCM’s reliance on metaphorical reasoning and imagery-based analogy fundamentally conflicts with Western analytical paradigms characterized by subject-object dualism and categorical classification. This divergence fosters misperceptions of TCM principles as irrational or fanciful, highlighting significant cultural and epistemological barriers in cross-cultural communication and knowledge transfer.
3	Cultural dissemination and adaptation	Balancing core values of traditional chinese medicine with local cultural contexts	The global dissemination of TCM must adhere to fundamental principles: a holistic approach and syndrome differentiation and treatment.
		The Principal contradictions in cultural dissemination and adaptation	Achieving a dynamic equilibrium between traditional paradigms and contemporary scientific expression necessitates a “translation reconstruction—narrative integration—systemic safeguarding” methodology.
4	Learning difficulties and conflicts	Theoretical study	Deciphering abstract theoretical constructs within TCM presents considerable challenges.
		Translation practice	Many core terminologies lack direct cultural equivalents in English, complicating literal translation and risking misinterpretation of the philosophical essence of dynamic balance inherent in TCM. When translating classical texts, it is imperative to thoroughly comprehend the profound meanings embedded in ancient writings and to render these into English expressions consistent with modern biomedical paradigms. This process involves elucidating the intrinsic connotations of key terms and translating them into contemporary biomedical frameworks.
5	Recommendations for resolution	Management leadership	Establishing an internationally recognized certification system for core competencies in TCM and fostering innovative collaborative mechanisms are essential steps.
		Individual competencies	Educational institutions should continue developing international faculty training programs, assemble interdisciplinary teaching teams, and coordinate educational resources to enhance cross-cultural communication skills and comparative understanding of Chinese and Western medicine. Curriculum development must reinforce the humanistic and philosophical foundations of TCM, integrating cross-cultural adaptation training and international communication strategies. Pedagogical methodologies can leverage intelligent teaching tools, digital platforms, and supplementary resources such as micro-lectures and Massive Open Online Courses (MOOCs). A multi-tiered collaborative effort among universities, educators, and practitioners is vital to effectively address the practical challenges associated with the international promotion of TCM.

### 4.3 Results analysis

#### 4.3.1 Micro-level perspective

Through the integration of qualitative interview data and subsequent three-phase coding analysis, a comprehensive understanding has been achieved concerning the multifaceted barriers encountered in the global dissemination of Traditional Chinese Medicine culture.

Firstly, from a theoretical standpoint, scholars recognize that: “*TCM emphasizes the systemic interconnection between humans and the natural environment, as well as the dynamic interplay of internal and external pathogenic factors influencing physiological homeostasis. It prioritizes subjective experiential knowledge and abstract conceptual frameworks such as ‘Qi, blood, Yin-Yang, and the Five Elements’, which are predominantly grounded in empirical traditional evidence, Taoist philosophical doctrines, and clinical praxis, reflecting a comprehensive holistic paradigm. Conversely, contemporary scientific epistemology advocates for objective empirical inquiry and data-driven validation, positing that pathological states can be elucidated through specific biochemical pathways and reproducible mechanistic models, emphasizing verifiability, reproducibility, and quantifiability. Reconciling the cultural epistemic foundations of TCM with the rigors of international scientific standards—acknowledging its pattern differentiation and therapeutic principles while aligning with Western biomedical research methodologies—constitutes a critical challenge in global medical education*”(S04). This underscores the fundamental dichotomy between TCM’s holistic, pattern-oriented conceptual framework and the reductionist, mechanistic approach characteristic of modern biomedicine.

Secondly, from a cross-cultural communication perspective, practitioners assert that: “*The traditional epistemological foundations underpinning TCM encounter significant barriers in international knowledge transfer. For example, the cosmological principle of ‘Unity of Heaven and Humanity ‘presents substantial interpretive challenges for Western audiences, who often find it difficult to comprehend why TCM emphasizes practices such as ‘nourishing yang during spring and summer, and nourishing yin during autumn and winter.’ Western biomedicine primarily concentrates on pathogenic agents, whereas TCM emphasizes the dynamic interaction between the human organism and its environmental context. The fundamental divergence stems from contrasting cognitive frameworks: TCM’s reliance on metaphorical analogies—such as comparing qi and blood circulation to water currents—is intuitive within holistic thinking cultures but is perceived as ‘unscientific’ by Western biomedical paradigms that prioritize logical empiricism*”(S04). This underscores the necessity of addressing cultural conceptual differences in the translation of TCM terminology to facilitate effective international dissemination. Semantic negotiation and culturally adaptive localization are thus essential for overcoming cognitive dissonance and enhancing cross-cultural acceptance.

Lastly, from a scientific perspective, experts underscore that overcoming terminological discrepancies, cultural biases, and commercial marginalization are essential strategies for the effective integration of “TCM” *within modern healthcare systems. They advocate that the global dissemination of TCM must adhere to fundamental principles such as holistic diagnosis and syndrome differentiation, while addressing three primary challenges: terminological inconsistencies—by implementing standardized translations aligned with international Chinese medicine nomenclature and utilizing visual representations of philosophical constructs; cultural biases—by integrating evidence-based acupuncture research within scientific discourse and leveraging community health narratives to foster local engagement; and commercial marginalization—by establishing ISO-compliant international standards and ethical review protocols. Achieving a dynamic equilibrium between traditional core concepts and contemporary scientific expression through translation standardization, narrative synthesis, and institutional regulation facilitates TCM’s role as an effective conduit for intercultural medical dialogue*.” This methodology demonstrates that employing standardized translation frameworks in practical applications diminishes comprehension and acceptance barriers, thereby promoting the broader dissemination of TCM principles.

This analysis identifies three primary challenges hindering the global dissemination of TCM: (1) Theoretical Disparities-Incompatibility of Paradigms. Respondents uniformly emphasize a fundamental epistemological divergence between TCM’s holistic diagnostic framework and the evidence-based reductionist approach of contemporary biomedicine. TCM employs abstract constructs such as Qi, blood, Yin-Yang, and the Five Elements, alongside clinical experiential knowledge, emphasizing dynamic interactions between humans and nature, as well as between form and spirit within spatiotemporal contexts. Conversely, modern medicine necessitates quantifiable, reproducible, and falsifiable biochemical mechanisms. The conceptual and evidentiary incongruence between these paradigms hampers integration in educational and research settings. (2) Cultural Divergences-Metaphorical Reasoning Divide. The principal obstacle in cross-cultural transmission arises from divergent cognitive models. TCM’s cosmological philosophy of “Unity of Heaven and Humanity,” principles of seasonal regulation, and reliance on metaphorical reasoning through imagery are perceived as “unscientific” within Western medical discourse, which emphasizes logical analysis and pathogen-centric models. Respondents cite examples such as “nourishing Yang in spring and summer, nourishing Yin in autumn and winter,” and the metaphor of water flow representing Qi and blood, highlighting the lack of experiential familiarity with these concepts in Western educational contexts, leading to interpretative dissonance. (3) Adaptive Challenges-Terminological Dilution and Commercial Exploitation. Linguistically, direct translation of traditional terminology often strips terms of their philosophical significance, resulting in semantic imbalance. Market-driven factors, including excessive commercialization, dilute the core values of TCM. Respondents categorize these issues as “cultural discount” and “commercial alienation,” both of which undermine the credibility and legitimacy of TCM within the global health community. In summary, disparities in cultural understanding and the necessity for contextual adaptation constitute the principal barriers to the effective international promotion of TCM culture.

#### 4.3.2 Meso-level perspective

The effectiveness of the international dissemination of traditional Chinese medicine hinges on the presence of a high-caliber, interdisciplinary, and versatile professional workforce.

The faculty members contend that: “*current pedagogical staff lack comprehensive expertise in both TCM and intercultural instructional methodologies, predominantly relying on conventional didactic approaches. In English-language TCM scholarly texts, translation efforts predominantly remain at a literal level, and the domain of TCM translation studies continues to evolve. The dual transmission of clinical content alongside cultural annotations presents a substantial pedagogical challenge. Additionally, the clinical training environment for multilingual and multicultural education is insufficient, as most case studies and clinical exemplars are regionally confined, impeding the global dissemination of Chinese medicine*” (T02). The instructors’ limited disciplinary backgrounds impose inherent restrictions on pedagogical strategies and knowledge transfer. The capacity of the faculty to deliver internationally oriented: “*TCM education is relatively underdeveloped; the current teaching cadre primarily comprises language specialists or practitioners with strong linguistic proficiency and Chinese medicine expertise. However, there is a notable deficiency of educators with specialized skills in intercultural communication, and the availability of teaching resources—including appropriate textbooks and digital learning materials—is limited*” (T03).

The research findings identify a tripartite fragmentation within the cadre of educators tasked with the international dissemination of TCM. Specifically, a disjunction exists between subject matter expertise and cross-cultural pedagogical methodologies, a disconnect between conventional instructional techniques and digital pedagogical modalities, and a misalignment between localized internationalization initiatives and global demand. This triad of issues precipitates systemic challenges, including a shortage of multidisciplinary educators possessing both TCM proficiency and intercultural communication competencies, reliance on monolithic pedagogical models, resource limitations, and constrained pedagogical capacity. The current workforce predominantly comprises individuals with singular linguistic or medical specializations, with traditional lecture-based approaches remaining predominant. There is a marked deficiency in immersive, multilingual, and multicultural training environments, as well as in the adaptation of international case studies. Furthermore, existing English-language educational materials are limited to literal translations lacking dual annotations that integrate medical and cultural contexts, compounded by a scarcity of supporting digital resources. Disciplinary constraints among educators impede the integration of language translation, cultural interpretation, and evidence-based communication within teaching practices. These limitations significantly hinder the depth and scope of TCM’s international outreach, representing a primary factor in the suboptimal outcomes of its global dissemination efforts.

#### 4.3.3 Macro-level perspective

The global acknowledgment of TCM remains constrained, necessitating immediate rectification of its professional standing within the healthcare sector. In scholarly literature, TCM is frequently categorized as complementary or alternative medicine, which engenders conceptual ambiguity and suggests a lack of integration within mainstream biomedical paradigms. The successful international dissemination and integration of TCM hinge upon the collaborative engagement of diverse stakeholders, including researchers, policymakers, and healthcare practitioners.

Experts have noted that:” international *qualification standards generally require practitioners of traditional Chinese medicine to possess an integrated knowledge framework encompassing both Chinese and Western medical principles. However, domestic training systems remain predominantly centered on traditional Chinese medical theories, with insufficient emphasis on Western medical fundamentals, resulting in a significant disconnect in curriculum alignment. The current curriculum emphasizes the purity of “Chinese medical thinking,” but fails to adequately develop the “Western medical instrumental skills” necessary for international accreditation—such as interpreting laboratory results and collaborating with Western medical practitioners. Consequently, students seeking certification often need to undertake extensive supplementary Western medical coursework or are compelled to retake certain Western medical subjects*”(S04). The analysis underscores that in the practical development of globally competent traditional Chinese medicine practitioners, there exists a disconnect between standardized pedagogical frameworks and real-world industry standards, leading to a curriculum misaligned with current market needs.

Students also observed: “*The existing curriculum sufficiently meets pedagogical requirements regarding cognitive load and instructional tempo. The program establishes a robust epistemological framework by allocating substantial instructional time to systematically elucidate the ‘standard English translations’ of Traditional Chinese Medicine terminology. Nonetheless, effectively conveying TCM concepts to international learners lacking prior foundational knowledge remains a considerable pedagogical challenge. Although the theoretical modules underscore the significance of intercultural communication, practical application opportunities are scarce and challenging to incorporate into authentic clinical or community contexts.*”(S03) Indicating that in routine pedagogical practice, educators tend to prioritize the translation of terminology, while in-depth interpretation and instruction of traditional Chinese medicine’s conceptual framework are often neglected, resulting in diminished efficacy of the international dissemination of TCM knowledge.

The interviewed scholars also observed that: “*the core contradiction between standardized curricula and individualized clinical instruction resides in the tension between the universality of didactic knowledge dissemination and the contextual specificity of clinical reasoning. Standardization guarantees the systematic evaluation of fundamental theories but may compromise the integrative, holistic approach inherent in syndrome differentiation and treatment modalities. Conversely, experiential learning through mentorship and case-based discussions enhances clinical acumen but encounters scalability limitations. Achieving pedagogical coherence necessitates adherence to Traditional Chinese Medicine principles: establishing foundational norms via standardization, complemented by the development of an open-access clinical case repository. In clinical education, integrating artificial intelligence—such as modular scenario simulations that combine virtual patient models with real case data—can support the development of a dynamic competency assessment framework, thereby balancing comprehensive knowledge acquisition with critical thinking skills essential for cultivating internationalized TCM practitioners*”(S02). This strategy also advocates for specific measures to align educational content with practical clinical requirements.

Based on multi-stakeholder interviews, the globalization of TCM talent development is currently hindered by a disconnect between accreditation standards, industry requirements, and clinical practice environments. International certification frameworks predominantly prioritize the integration of Chinese and Western medical competencies; however, domestic curricula remain predominantly rooted in traditional TCM theories, with limited emphasis on practical Western medical skills. Consequently, students often need to undertake additional or remedial Western medical training. While classroom instruction systematically addresses English medical terminology translation, it lacks immersive, scenario-based training that effectively conveys core concepts to learners without prior TCM knowledge, resulting in a gap between translation ability and clinical communication proficiency. Standardized pedagogical approaches ensure theoretical rigor and assessment but fail to reflect the dynamic nature of clinical reasoning and syndrome differentiation. Furthermore, mentorship-driven clinical observation and case discussions face scalability challenges. Fundamentally, there exists a misalignment between certification standards, healthcare market demands, educational methodologies, and real-world clinical contexts. Addressing these issues necessitates the creation of an open, multilingual medical case database integrated with AI-powered modular scenario simulations and a dynamic evaluation system to bridge the divide between universal knowledge dissemination and context-specific clinical cognition.

In summary, analyses at the micro, meso, and macro levels reveal that “cultural paradigm conflict” to some extent limits the international dissemination and recognition of TCM. However, relying solely on this explanation fails to fully reveal the complex reasons for the low international acceptance of TCM. The incompleteness of the clinical evidence system and barriers to entry in the international medical market are also key constraints. First, from an evidence system perspective, TCM lacks sufficient evidence-based data support within the mainstream international medical system (49). Its research largely remains at the case or experience level, failing to meet the stringent requirements of Western evidence-based medicine for randomized controlled trials and standardized data. This “evidence gap” weakens TCM’s voice in the global academic and regulatory systems. Second, at the international medical market level, significant differences in regulatory systems, drug registration standards, and professional entry barriers across countries create multiple obstacles for TCM products and services in areas such as certification, insurance reimbursement, and legal practice. For example, some countries still categorize TCM as alternative or complementary medicine, limiting its clinical application and professional status.

### 4.4 Fundamental origins of the central conundrum

#### 4.4.1 Cultural paradigm conflict

The dissemination of TCM within the framework of globalization is impeded by fundamental structural factors rooted in cultural discontinuities and epistemological alienation. As a life sciences discipline grounded in China’s indigenous knowledge system, TCM constructs a unique ontological worldview of the human body, pathogenesis, and therapeutic intervention that diverges from contemporary biomedicine. Its core paradigms-holism and syndrome differentiation-embody a “unity of heaven and humanity” cognitive model.

However, the developmental lag in theoretical innovation has emerged as a critical bottleneck hindering its international integration. Higher education institutions face constraints due to the multifaceted demands of pedagogy, research, and clinical practice, limiting sustained engagement with advanced theoretical development and innovation. Concurrently, clinical practitioners in TCM hospitals are subjected to dual pressures of intensive diagnostic and therapeutic workloads alongside prolonged research cycles, which restrict the scope and depth of scholarly inquiry. More critically, the subjective and ambiguous nature of TCM theories and methodologies-compared to the objective framework of Western medicine, which relies on quantifiable biomarkers and chemical analysis-often leads to perceptions of a lack of empirical verifiability and scientific legitimacy within modern scientific discourse. These factors further reinforce the cognitive barriers to cross-cultural transmission and global acceptance of TCM.

The educator emphasized that: “*the primary contradiction in the global dissemination of TCM education pertains to disparities in cultural literacy and conflicting epistemological frameworks. International students frequently lack a profound understanding of the cultural and philosophical foundations underlying TCM theories. While English translations facilitate literal interpretation, they often fail to encapsulate the nuanced metaphysical concepts such as ‘Yin-Yang’, 'Five Elements’, and ‘Qi’, thereby obstructing a comprehensive understanding of their intrinsic philosophical significance. Additionally, the dominance of empirical validation and linear causality in Western biomedical paradigms leads to divergent methodologies in holistic pattern differentiation within TCM. Students trained within Western medical epistemologies tend to interpret TCM through this familiar scientific lens, which impairs their authentic grasp of its integrative diagnostic and therapeutic principles. These discrepancies extend beyond linguistic translation, revealing fundamental philosophical and cultural comprehension challenges in the internationalization of TCM educationt*” (T01). This further demonstrates that cultural misunderstandings and conflicts in cognitive frameworks significantly impact the effectiveness of the international dissemination of traditional Chinese medicine culture.

Traditional Chinese medical texts and terminology are often obscure and complex, encompassing rich and intricate concepts such as Yin-Yang theory, the Five Elements, meridians and Qi-Blood dynamics, and the functional relationships of zang-fu organs. The contextual depth and cultural nuances pose significant challenges for accurate translation and adaptation, leading to considerable debate within the field. Although multiple translations of classical texts exist, their quality varies considerably. The fidelity and scholarly rigor of these translations directly influence the effectiveness of international dissemination of Traditional Chinese Medicine (TCM) and its cultural heritage, thereby constraining the global integration and recognition of TCM practices. The student emphasized that: “*the primary obstacle in translating Traditional Chinese Medicine (TCM) lies in overcoming the cultural and conceptual translation barrier associated with its abstract terminologies. Many fundamental TCM terms lack direct cultural equivalents in English, complicating literal translation and increasing the risk of misrepresenting the underlying philosophical principle of dynamic equilibrium. When translating classical TCM texts, it is crucial to comprehend the profound meaning of ancient doctrines and adapt them into contemporary medical logical expressions in English. In practical applications, consideration of the recipient’s medical literacy level is essential to accurately convey complex concepts, thereby critically evaluating the translator’s capacity for adaptive and spontaneous response*” (T03). Scholars engaged in the international dissemination of TCM culture often possess a narrow disciplinary background and lack comprehensive cross-cultural training, which constrains the global communication and exchange of TCM cultural knowledge.

#### 4.4.2 Challenges in teaching

The shortage of dual-qualified educators who possess foundational knowledge of TCM theory, demonstrate strong foreign language proficiency, and are well-versed in international cultural contexts and cross-cultural communication skills is also a significant factor affecting the effectiveness of the international dissemination of TCM. The educators noted that: “*the faculty capable of delivering internationalized TCM instruction is relatively underdeveloped. The current teaching cadre predominantly comprises either language specialists or educators with a background in TCM who possess strong linguistic skills. However, there is a scarcity of instructors proficient in communication strategies specific to TCM dissemination. Additionally, the instructional process is hindered by a lack of tailored textbooks and digital resources, resulting in a significant deficiency of teaching materials*” (T03). The analysis further underscores that teachers’ limited disciplinary expertise and scarcity of teaching resources undermine their pedagogical efficacy. “*Regarding the globalization of traditional Chinese medicine education, faculty members exhibit insufficient foreign language competencies, limited international training experience, and a paucity of qualified instructors. Concerning curricular resources, there is an abundance of heterogeneous and inconsistent editions, with a notable absence of standardized international textbooks. In terms of educational infrastructure, overseas institutions are hindered by suboptimal facilities and a shortage of clinical internship sites, while domestic institutions’ infrastructure also falls short of meeting the requirements for internationalized medical education*” (T04). This also elucidates the paucity of pedagogical resources, the inadequate foreign language proficiency of professionally trained educators with medical backgrounds, the deficiency of overseas clinical internship sites, and significant challenges in the practical training component for cultivating talents in the international dissemination of TCM.

Domestic students interviewed reported that: “*the curriculum is comprehensive; however, the elevated cognitive load and accelerated instructional tempo impede in-depth comprehension. They also identified a disjunction between theoretical instruction and practical application, with limited experiential learning opportunities*” (S06). “*Although the program generally fulfills their academic requirements, the practical training component remains underdeveloped, predominantly comprising didactic lectures on traditional Chinese medicine principles and dissemination methodologies. There is a conspicuous deficiency of experiential training aligned with authentic international communication contexts, such as simulated scenarios for Chinese medicine science popularization abroad or strategies to mitigate cross-cultural skepticism*” (S13). Both respondents concurrently indicated that the pedagogical curriculum framework inadequately integrates theoretical constructs with practical application, lacking authentic contextual experiential learning and neglecting the domestication of international communication processes. This deficiency impedes students’ capacity to undergo a qualitative transformation from cognitive acquisition to skill mastery, resulting in ineffective knowledge transfer and consequently compromising instructional efficacy and the overall learning experience.

#### 4.4.3 The challenge of standardization

In recent years, TCM has increasingly become a prominent symbol of Chinese culture’s global outreach, with significant progress in its internationalization. However, challenges remain in standardizing TCM education. The framework for modern discourse and authoritative systems governing traditional Chinese medicine has yet to be fully established. Although the unique therapeutic effects and scientific connotations of TCM are gradually gaining international recognition and acceptance as a distinct medical science, many overseas populations still find it difficult to comprehend and accept the core philosophical concept of “harmony between heaven and man” intrinsic to TCM culture. Additionally, there is a lack of uniformity in TCM education and practice standards across different countries.

Scholars contend that: “*the primary conflicts and integration challenges between international certification standards for TCM practitioners—such as educational background, language proficiency, and practical requirements—and the domestic training system lie in the recognition of educational credentials and practical training. Overseas standards often emphasize credit hours, specific course modules, and clinical internship hours, sometimes underestimating the depth of classical TCM theory study and the uniqueness of apprentice-based practice. Conversely, students trained domestically frequently exhibit deficiencies in language skills and familiarity with local healthcare regulations and insurance systems. This disparity can be likened to incompatible operating systems with incompatible interfaces. For instance, the mentorship experience emphasized domestically is difficult to quantify and evaluate within foreign certification frameworks*” (S03). “*International qualification standards generally require practitioners of TCM to possess an integrated knowledge framework encompassing both Chinese and Western medical principles. However, domestic training systems remain predominantly centered on classical TCM theories, with insufficient emphasis on Western medical fundamentals, resulting in a significant disconnect in curriculum alignment. The current curriculum emphasizes the purity of “Chinese medical thinking”, while neglecting the development of ‘Western medical instrumental skills’ essential for international accreditation—such as interpreting laboratory reports and collaborative diagnosis with Western medicine. Consequently, students seeking certification are often compelled to undertake extensive supplementary Western medical coursework or even repeat certain Western medical subjects, thereby impeding seamless integration into internationally recognized standards*.” (S04) Scholars have noted significant differences in TCM education worldwide, challenges in its adaptability. And its relatively low recognition in healthcare and science.

#### 4.4.4 The challenge of students

Language barriers, disparities in learning motivation, cultural shock and adaptation difficulties, and unclear future career pathways represent significant challenges faced by students. The international dissemination of traditional Chinese medicine suffers from a shortage of interdisciplinary, versatile talent pools, with inconsistent training quality. Consequently, students encounter various confusions and obstacles throughout their learning process.

The student remarked, “*The most challenging aspect is the dual translation of specialized terminology: first, understanding classical Chinese medical texts such as the ‘Ying Wei’ concept in the ‘Huangdi Neijing’, and then translating it into English that aligns with Western medical paradigms. For instance, ‘Bian Zheng Lun Zhi’ must be rendered differently depending on the audience: for scholars, ‘syndrome differentiation and treatment,’ whereas for the general public, it should be simplified to ‘pattern-based personalized therapy.’ This process demands a high level of linguistic precision and medical expertise.*” (S01) "The dual translation challenge of TCM terminology: first requiring an understanding of Chinese medical principles, then translating into English pedagogical language. For example, when explaining ‘Qi deficiency’, it is essential to avoid the misleading direct translation as ‘energy deficiency’.” (S011) Both students noted that the primary obstacle in the learning process stems from the cultural and cognitive differences between Chinese and Western thinking paradigms, which create significant linguistic barriers. This is particularly evident in the translation of specialized terminology, where it is essential to first comprehend the cultural connotations of TCM before rendering accurate and contextually appropriate English equivalents.

However, learners demonstrate considerable heterogeneity in their intrinsic and extrinsic motivational constructs. “*The selection of the International Micro-Program in TCM dissemination stems from dual interests: firstly, the potential application of TCM in global health, exemplified by evidence-based research on acupuncture for pain management; secondly, a sense of mission to break down cultural barriers. The primary objectives include: (1) mastering precise English terminology for TCM; (2) understanding the compliance requirements of international healthcare systems regarding TCM; and (3) enhancing cross-cultural communication skills in clinical practice to prepare for future involvement in WHO traditional medicine initiatives*.” (S01) “*I aim to acquire a new skill related to my field to broaden my perspective. Primarily, I seek to articulate TCM terminology and principles clearly in professional English, enabling effective communication of its underlying cultural concepts to audiences from diverse cultural backgrounds. Additionally, in an era characterized by interdisciplinary integration, possessing skills beyond my core discipline offers a competitive advantage in both employment and academic pursuits*”. (S03) Both two students articulated their initial motivation for studying international communication of traditional Chinese medicine, emphasizing their personal interests and the potential to enhance employability through this specialized knowledge.

In the practical application, students also encounter certain real-world challenges. For instance, interviewees mentioned “*the issue of timeliness, specifically the need for prompt interpretation, where during consultations, traditional Chinese medicine diagnoses must be rapidly translated into the patient’s native language, demanding exceptionally high real-time responsiveness.*” (S01) “*Communication barriers exist when engaging with international students and patients speaking other languages. Prior to specializing in the international dissemination of Traditional Chinese Medicine (TCM), my proficiency in English technical terminology was limited, impeding effective communication with international students. For patients using different languages, the precise meanings of our English technical terms are often incomprehensible, and their connotations are lost when directly translated, making it challenging to provide clear and accessible explanations. In practical applications of TCM cultural dissemination.*” (S02) Students frequently encounter culture shock and face difficulties in accurately translating and conveying concepts in a manner that ensures mutual understanding.

Furthermore, students face challenges such as unclear career development pathways in the international dissemination of TCM. For instance, “*their goal is to engage in teaching or translation roles within international TCM training institutions. While current coursework provides a foundational knowledge base, its practical utility is moderate; students have studied TCM theories and foreign languages, yet there is limited instruction on integrating these disciplines to design curricula or address queries from international audiences. The training approach remains predominantly academic, with a noticeable gap from actual industry requirements. There is a pressing need to incorporate more industry-relevant content, such as understanding the operational models of international TCM organizations.*” (S12) It was mentioned that although the future career plan is to work in the field of international training of TCM, there is still a certain gap between the content of school learning and the actual work requirements, resulting in a mismatch between the students’ training goals and social needs.

Although TCM has been promoted to 196 countries and regions worldwide, due to issues with professional qualification certification between countries, overseas TCM colleges tend to train more localized talent, making it difficult for domestically trained students to find employment abroad. Furthermore, due to geographical factors, the internationalization of TCM in Beijing, Shanghai, and Guangzhou is higher than in western regions, resulting in fewer opportunities for students in western regions to interact with overseas TCM learners, leading to fewer job opportunities related to the international dissemination of TCM (50). While employment paths for international TCM professionals are diverse, significant differences in required qualifications, policies, regulations, and career advancement mechanisms across various fields often leave students struggling to identify clear career development directions during their studies, creating a “path ambiguity” dilemma. This phenomenon highlights the need for improvement in career guidance and international curriculum design by universities and policymakers.

#### 4.4.5 The challenge of resource and policy dilemmas

Currently, the imbalance in talent development supply and insufficient funding are also primary factors affecting the effectiveness of international dissemination of TCM. “*The primary challenge lies in the uneven resource allocation and conflicting interests. The government aims to rapidly expand influence, universities focus on disciplinary assessments and publication output, the market demands immediately profitable talent, and overseas partners often prioritize short-term project gains. Consequently, activities requiring long-term investment and foundational work-such as the development of high-level bilingual textbooks, the establishment of overseas clinical teaching bases, and cross-cultural faculty training-are frequently overlooked. All parties tend to prefer harvesting immediate benefits rather than planting seeds for future growth, making coordination exceedingly difficult.*”(S03) "*I believe that when coordinating multiple stakeholders in the internationalization of talent development, the core contradictions and challenges primarily revolve around the following aspects: firstly, misaligned goal positioning, leading to conflicting interests; the government emphasizes talent cultivation and strategic reserves, whereas universities focus more on enhancing their reputation and ranking. Consequently, the government pursues long-term benefits for talent reserves and social welfare, while universities prioritize academic prestige and status. Secondly, resource allocation disparities are evident; the government concentrates on macro-level planning, investing predominantly in projects with strict cyclical constraints, whereas universities tend to allocate resources to their strengths in specific disciplines, aiming for short-term returns.*” (S05) In expert interviews, it was consistently noted that universities and government agencies display conflicting objectives and demands in talent cultivation, with resource disparities limiting the development of professionals for TCM internationalization and impeding its global integration. Specifically, there is a misalignment between universities and the government in their goals and needs for cultivating international TCM talent. Universities prioritize academic training and research outcomes from international collaborative projects, while government departments emphasize policy guidance and practical effectiveness in serving national strategies. This difference in goals directly leads to inconsistencies in resource allocation and priority areas in the short term. Further analysis reveals that insufficient resource allocation is neither simply a matter of funding shortages nor an occasional implementation deviation, but rather exists simultaneously at both the institutional design and policy implementation levels. In terms of institutional design, the lack of a top-level coordination mechanism oriented toward collaborative governance results in a lack of integration and long-term planning of investment goals across different departments. At the policy implementation level, due to overlapping responsibilities and imperfect coordination mechanisms between departments, resource allocation often results in delays, duplication, or inefficient use when implemented in universities or overseas projects. This dual imbalance between system and implementation constrains the overall effectiveness of the TCM international communication talent training system and weakens the policy’s sustained influence. To achieve truly collaborative advancement, it is urgent to improve the top-level design through institutional innovation and establish cross-departmental collaboration and dynamic evaluation mechanisms in the implementation phase, fundamentally optimizing the resource allocation logic.

#### 4.4.6 The dilemmas of international communication of TCM from multiple perspectives

While different groups have varying emphases in their understanding of the dilemmas of international communication of TCM, their concerns are not isolated but rather present a progressive logic, moving from “practical barriers” to “systematic issues in the knowledge system.” Students generally regard “language barriers” as the most direct difficulty, reflecting the comprehensibility and expressibility of TCM knowledge in cross-cultural contexts—a primary bottleneck in the “knowledge transformation” process. Teachers focus more on the localization and cultural adaptation of teaching content, demonstrating an exploration of knowledge translation strategies at the educational implementation level. Scholars and experts, however, elevate their focus to the levels of “interdisciplinary paradigms” and “institutional synergy,” believing that the fundamental challenge of international communication of TCM lies not only in the language level but also in how to redefine the theoretical legitimacy and methodological status of TCM within the scientific discourse system. This hierarchical difference in perspectives reflects the multidimensional dilemmas of international communication of TCM, ranging from micro-level language and teaching practices to meso-level educational systems and disciplinary interactions, and finally to macro-level knowledge systems and international governance structures. Understanding the intrinsic connections between these levels helps to develop more integrated response strategies at the theoretical and policy levels, and to achieve a transformation from “language interoperability” to “knowledge co-construction.”

## 5 Discussion

### 5.1 Comparative analysis of this study and prior research

Previous scholarly investigations have predominantly identified cultural disparities and variations in cognitive paradigms between Chinese and Western contexts as pivotal factors impeding the global dissemination of traditional Chinese medicine (TCM) (49). The present study substantiates these findings, with consensus among participants indicating that cultural discontinuities and cognitive alienation serve as primary determinants influencing the efficacy of international communication (50, 51). The cultural and cognitive barriers emphasized in earlier research are robustly validated within this investigation, aligning with established literature.

Nevertheless, this research uncovers notable divergences in the focal points among different participant cohorts concerning the challenges associated with TCM’s international propagation (Table 5). Compared to existing international studies, this research underscores its distinctiveness and innovative approach. Specifically, students and educators predominantly address linguistic and conceptual obstacles encountered at the pedagogical interface. Conversely, scholars and domain experts prioritize issues related to the inadequacy of interdisciplinary paradigm integration and the deficiencies within institutionalized training pathways.

**Table 5:** Differences between existing studies and this research.

NO.	Type	Existing research	This study
1	Research subject	The focus predominantly lies on translation studies, linguistics, or clinical efficacy perspectives, neglecting the diverse roles of educators, students, and experts.	Presenting the differentiated concerns of multiple stakeholders—students, educators, researchers, and specialists—through 31 cross-sector interviews.
2	Core issues	Emphasis is placed on the accuracy of terminology translation and validation of clinical outcomes.	Elucidating the interactive mechanisms underlying the triadic dilemmas of linguistic, pedagogical, and institutional frameworks across micro, meso, and macro levels.
3	Theoretical framework	The approach primarily relies on a singular framework of translation studies and medical education.	Integrating theories of cultural mediation, knowledge transfer, and collaborative governance to propose a dual-track model of knowledge dissemination combined with participatory governance.
4	Research methodology	With literature reviews and case studies as the main methodologies.	Utilizing semi-structured in-depth interviews and a three-tiered coding scheme to develop comprehensive cross-group evidence.
5	Cross-cultural perspective	The dissemination of traditional Chinese medicine is viewed as a unidirectional transfer of knowledge, lacking consideration of cognitive construction and cultural negotiation.	Focusing on semantic negotiation and cultural adaptation, with particular emphasis on identity recognition and communicative self-efficacy among intercultural learners.
6	Practical implications	The discourse is heavily centered on academic discussion, with insufficient development of systematic policy and educational recommendations.	Recommending practical interventions such as interdisciplinary faculty development programs and standardized certification systems. Adhering to the principle of “gradual reform,” and on the basis of respecting and absorbing existing certification achievements, we will promote the upgrading and integration of the standards for core competencies of traditional Chinese medicine.

Furthermore, the roles assumed by individuals in the international dissemination process exert a more significant influence in this context. Educators and students tend to emphasize tangible pedagogical challenges, such as cultural translation barriers of abstract TCM concepts—many core terminologies lack culturally equivalent English counterparts, complicating direct translation and increasing the risk of misinterpretation of TCM’s underlying philosophy of dynamic balance. In contrast, scholars and experts focus on issues pertaining to the integration of interdisciplinary cognitive frameworks, the practical demands of international development, and the limitations of current talent cultivation systems.

These findings imply that the international promotion of TCM extends beyond linguistic and cultural barriers, encompassing group-level cognitive differences and institutional structures. Accordingly, this study introduces an innovative “dual-track knowledge translation and collaborative governance” framework, providing a novel theoretical perspective for understanding the multidimensional challenges faced in TCM’s global dissemination and offering strategic insights for optimizing future talent development models.

### 5.2 Causes and determinants of variance

The analysis indicates that the primary source of observed discrepancies stems from the conflict between institutional paradigms and practical implementation within the internationalization of TCM higher education. Specifically, the rapid internationalization of TCM higher education, driven by policy initiatives such as the Belt and Road Initiative, has facilitated increased international exchanges and demand for global dissemination. Nonetheless, the development of human capital and educational infrastructure remains insufficient, with a limited pool of professionals skilled in international TCM communication. Prior research has predominantly concentrated on terminological accuracy and standardization, with limited focus on cultivating international communication competencies, curriculum development, instructional content, and educational resources such as textbooks (52). This study employs a multi-tiered analytical framework encompassing Micro-Meso-Macro.

At the micro-level, semantic negotiation and strategic decision-making by communicators within specific intercultural contexts underscore the embedded “practical wisdom” in cross-cultural discourse. At the meso-level, pedagogical reforms by educators illustrate the reproduction of discourse systems during intercultural translation processes. At the macro-level, strategic policy adjustments and interdisciplinary paradigm selection reflect the structural tensions between educational systems and internationalization imperatives. The integration of this layered framework facilitates a comprehensive understanding of TCM’s international dissemination within a complex “language-education-system” domain, thereby advancing scholarly insights into dissemination mechanisms.

### 5.3 Limitations and future research

To address the challenges associated with cultivating international advocates for TCM, the qualitative insights obtained from stakeholder interviews provide both scholarly significance and practical implications. Nonetheless, several limitations are apparent. First, the scope of the research perspective is restricted. Although this study targeted students, educators, scholars, and experts, the sample remains predominantly localized, with an insufficient representation of international students, leading to conclusions that predominantly reflect a “localization bias” from a singular perspective. Second, the methodological approach exhibits constraints. The primary reliance on qualitative interviews, without substantial quantitative data to substantiate the findings, results in conclusions that are overly theoretical and adhere to a singular evaluative paradigm. Third, the research depth is limited. This study outlines the conflicts between different stakeholders but lacks in-depth analysis of the necessary connections between them, leading to an overly simplistic and singular analysis of the causes. In particular, the low international recognition of TCM should be understood as a result of the interplay of cultural paradigms, scientific evidence, and the institutional environment. Cultural differences influence cognitive acceptance, the evidence system affects scientific legitimacy, and market access affects the implementation of regulations. Only by simultaneously promoting the standardization of clinical research and the construction of international certification systems based on cross-cultural understanding can we truly overcome the systemic bottlenecks in the international dissemination of TCM.

These limitations underscore the need for future research to incorporate cultural variables more comprehensively and to broaden the diversity and geographic scope of interview participants across different nations. Future investigations could focus on several key areas: (1) Cross-cultural comparative analysis and empirical validation. Expanding samples to include international students and educators from diverse cultural backgrounds, and conducting transnational comparisons to elucidate how cultural discontinuities influence dissemination across various social contexts. (2) Mixed-methods research design. Integrating surveys, experimental methodologies, and big data analytics to generate more robust quantitative evidence regarding dissemination efficacy and cultural perceptions. (3) Interdisciplinary theoretical integration. Incorporating frameworks from communication studies, education, and sociology to mitigate the current over-reliance on translation and linguistics paradigms. (4) Policy implications and practical applications. Developing actionable strategies for talent development and international curriculum design from a collaborative governance perspective, thereby enhancing the linkage between academic research and practical implementation.

In conclusion, this study not only affirms the constraining effects of cultural differences and cognitive alienation on the global dissemination of TCM but also uncovers group-specific concerns and systemic tensions. It provides a comprehensive analytical framework for academia and offers empirical insights for policymakers and educational practitioners.

## 6 Conclusion

### 6.1 Key findings

Participants from four stakeholder groups-students, educators, domain experts, and scholars—generally agree that the primary barriers to the global dissemination of TCM are rooted in cultural discontinuities and cognitive dissonance. Students primarily identify challenges in the translation of TCM terminology and cultural adaptation; educators emphasize the conflict arising from divergent cultural paradigms and cognitive frameworks. Students demonstrate a superficial understanding of the cultural and theoretical foundations intrinsic to TCM, with English translations often limited to literal renderings. Scholars contend that the obstacles to TCM’s internationalization stem from epistemological and methodological disparities between TCM knowledge systems and contemporary biomedicine. Experts note that domestic TCM education has historically been tailored to local healthcare needs, resulting in standardized curricula that lack adaptation for diverse international medical systems. Additionally, cross-cultural acceptance of TCM varies significantly, further impeding its global integration. The current educational infrastructure inadequately addresses these issues, as students often lack intercultural communication competencies and familiarity with international regulatory frameworks, which may lead to ethical conflicts arising from cultural misinterpretations during overseas practice. Analysis of interview data indicates that the internationalization of TCM fundamentally involves a contest over discursive sovereignty between Eastern and Western civilizations, with policy contradictions revealing a core tension between the push for standardization and the imperative to preserve cultural distinctiveness. These significant findings further substantiate the research questions RQ1, RQ2 and RQ3.

### 6.2 Significance of research and policy recommendations

The international dissemination of TCM culture is a gradual process necessitating sustained efforts in talent development, involving coordinated strategies among governmental agencies, academic institutions, and industry stakeholders to enhance the global promotion of TCM.

Firstly, it is imperative to strengthen the strategic framework for TCM international communication. Governments should actively foster collaboration among universities, research institutes, TCM higher education institutions, and transnational enterprises, establishing a synergistic innovation ecosystem for the global dissemination of TCM culture. Strategic planning for international outreach should be formulated, including the establishment of an “International TCM Talent Development Consortium.” Project-based initiatives should facilitate substantive partnerships with overseas TCM organizations, clearly defining stakeholder responsibilities, rights, and benefits to promote resource sharing, complementary strengths, and collaborative innovation. Additionally, increased financial investment is essential, including dedicated funding for the development of overseas educational facilities, clinical centers, and cultural exchange platforms, thereby providing a robust infrastructural foundation for the international promotion of TCM.

Secondly, efforts must be intensified to cultivate specialized talent for the global dissemination of TCM. The World Health Organization’s “Global Traditional Medicine Strategy 2025–2034” underscores the importance of safeguarding traditional medicine rights and interests (53). Higher education institutions, as primary talent cultivation platforms, should leverage their core functions by designing targeted curricula and training programs aimed at developing international communication competencies in TCM (54). This includes establishing micro-specializations such as International TCM Communication Studies, offering courses on TCM culture and its global dissemination, and enabling students to comprehend TCM’s fundamental philosophies and cultural richness. Cross-national collaboration among TCM universities should be pursued to overcome regional barriers, developing multilingual and cross-cultural pedagogical resources. Platforms should be established to expand faculty internationalization training and exchange opportunities. Furthermore, students should receive systematic cross-cultural adaptation training and career development support.

Thirdly, it is essential to develop internationally recognized certification systems for core TCM competencies and to establish innovative, collaborative governance mechanisms. Building an internationally recognized certification system for core competencies in TCM should be based on integrating existing international frameworks, rather than completely rebuilding them. For example, the World Federation of Chinese Medicine Societies has launched the “International Certification System for TCM Practitioners,” providing a preliminary basis for international access and professional standards for TCM professionals. However, this system still faces problems such as insufficient regional applicability, inconsistent standards, and a relatively singular competency assessment dimension during its implementation. Therefore, this study recommends a “gradual reform” approach, respecting and incorporating existing certification achievements to promote the upgrading and integration of TCM core competency standards. Specifically, efforts should be concentrated on developing a set of education and competency standards that embody the essence of TCM (such as syndrome differentiation and treatment, and classical applications) and can be understood and quantitatively assessed by international peers, serving as a “bridge” and “common language” connecting certification systems in different countries. To ensure its sustainability and international influence, it is recommended to establish a multi-party collaborative platform led by a high-level coordinating body (such as the State Administration of Traditional Chinese Medicine), integrating resources from government departments (responsible for policy design and funding support), universities (responsible for talent cultivation and academic research), industry associations (responsible for standard setting and certification), and overseas institutions (providing localized implementation and feedback). Through co-construction, sharing, and gradual integration, an open, compatible, and sustainable international TCM certification ecosystem can be gradually formed, achieving a systematic leap from “single-point trials” to “global collaboration.”

## Data Availability

The original contributions presented in the study are included in the article/supplementary material, further inquiries can be directed to the corresponding authors.

## Appendix

### Interview topics

(1) Interview Theme:

Research on the International Dissemination and Education of Traditional Chinese Medicine (TCM).

(2) Interview Questions:

Question 1: During your direct teaching of students, what do you perceive as the most prominent and common contradictions? (Cultural understanding differences, language proficiency, cognitive style conflicts, learning goal disparities, insufficient teaching resources, etc.)

Question 2: The profound Chinese traditional cultural elements embedded in TCM (philosophy, cosmology, values) face deep-rooted cultural incommensurability or comprehension barriers when transmitted to learners from diverse cultural backgrounds. What are the core points of contradiction?

Question 3: How does the academic debate between "scientification of TCM" and "culturalization of TCM" influence the positioning of international talent cultivation objectives? How are these two approaches in conflict?

Question 4: In promoting the international dissemination of TCM culture, how can we avoid cultural misinterpretation, oversimplification, or vulgarization?

Question 5: How can we balance the core values of TCM with the need to adapt to local cultural contexts? What are the main contradictions involved?

Question 6: During your studies in the field of TCM international communication (theoretical learning, classical texts study, translation practice, etc.), what specific difficulties have you encountered? (Language barriers, understanding abstract concepts, memorizing numerous technical terms, etc.)

Question 7: Based on your experience, what effective strategies or methods have you employed to reconcile these contradictions? (Please provide specific examples.)

Question 8: In your opinion, at which levels (policy, university management, curriculum design, methods, personal capacity) should reforms or improvements be implemented to better address these contradictions?

Question 9: Regarding stakeholder collaboration (government, universities, organizations, overseas institutions), what are the current major obstacles? What suggestions do you have for improvement?

Question 10: Which levels (policy, university management, curriculum design, methods, personal capacity) require reform or enhancement to more effectively reconcile these contradictions?

Question 11: Based on your personal experience, in what aspects can universities, teachers, and administrators improve to assist students in the micro-specialty of TCM communication in overcoming learning difficulties and alleviating the contradictions you mentioned? (provide specific, recommendations.)
